# Supplementary figures and images for: Can physiological network mapping reveal pathophysiological insights into emerging diseases? Lessons from COVID-19
Source: PLoS One. 2025 Nov 21;20(11):e0337333. doi: 10.1371/journal.pone.0337333 (PMC12637946; doi:10.1371/journal.pone.0337333)

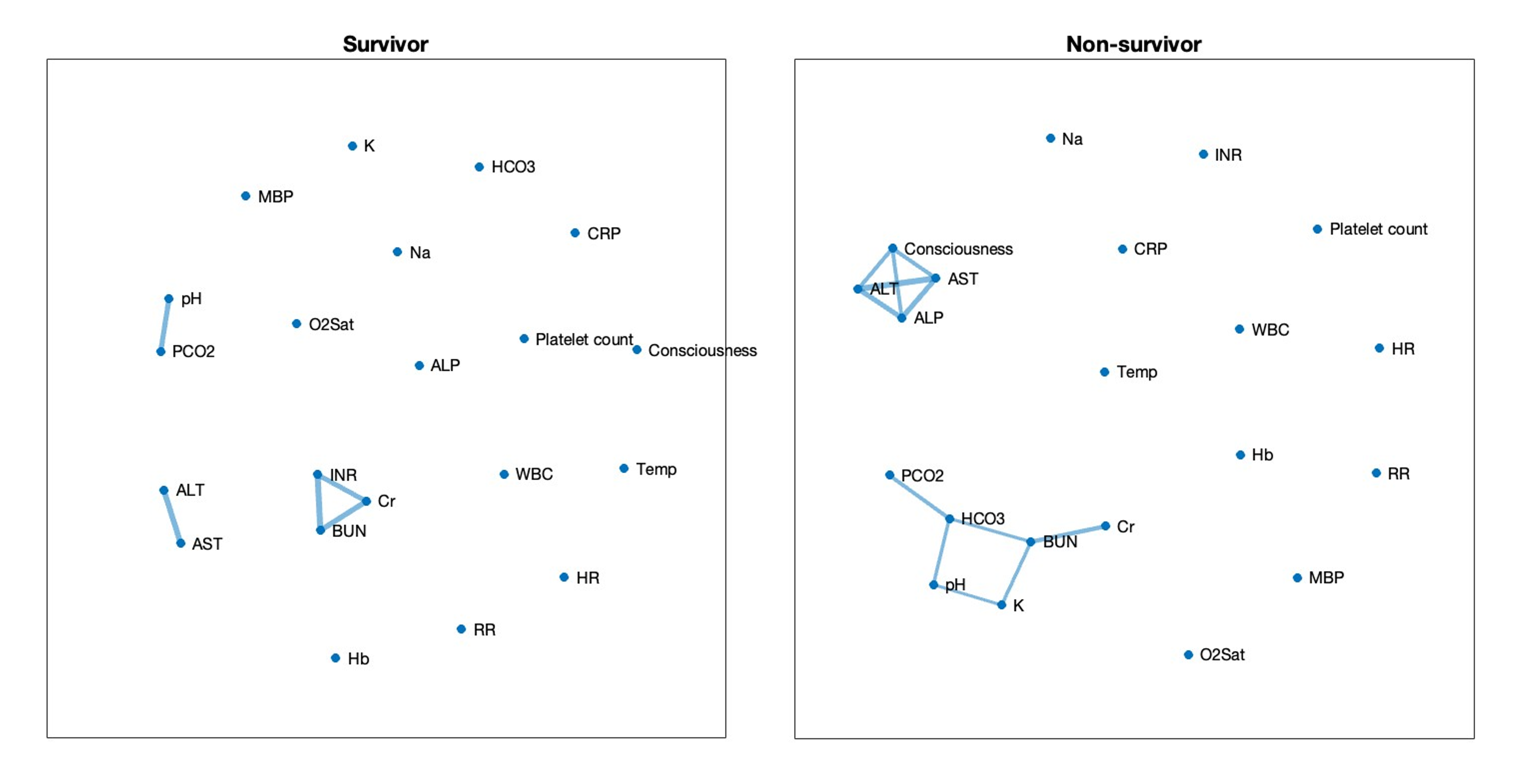

Supplement: S1 Fig — Matching for the groups was performed using an automated algorithm to pair patients according to their capillary oxygen saturation (O2Sat). Each link shows a statistically significant correlation between two biomarkers after Bonferroni correlation for the total number of multiple comparisons. Abbreviations: Hb – Haemoglobin; WBC – White Blood Cell count; Platelet counts – Platelet count; CRP – C-reactive protein; Cr – Serum creatinine; BUN – Blood urea nitrogen; AST – Aspartate aminotransferase; ALT – Alanine aminotransferase; ALP – Alkaline phosphatase; Na ⁺ – Sodium; K ⁺ – Potassium; INR – International normalized ratio; pH – Arterial hydrogen ion concentration; PCO2 – Arterial partial pressure of carbon dioxide; HCO3 – Arterial bicarbonate concentration. (TIF) [file pone.0337333.s001.tif]
