# Supplementary material for: Can physiological network mapping reveal pathophysiological insights into emerging diseases? Lessons from COVID-19
Source: PLoS One. 2025 Nov 21;20(11):e0337333. doi: 10.1371/journal.pone.0337333 (PMC12637946; doi:10.1371/journal.pone.0337333)
Supplement: S2 Table — (DOCX) [file pone.0337333.s003.docx]

**Supplementary S2 Table:** Principal Component Analysis (PCA) of Survivors and Non-Survivors Using Eigenvalue 2 as the Cut-Off

PCA was conducted following Varimax rotation and Kaiser normalisation. The Kaiser-Meyer-Olkin (KMO) test confirmed that the sample was adequate for PCA, with p-values < 0.001 (Chi-square = 625.456 for survivors and 537.345 for non-survivors; p < 0.001).

***Survivor***

|  | Principal Components | |
| --- | --- | --- |
| Variables | 1 | 2 |
| BUN | 0.859 |  |
| Cr | 0.78 |  |
| HCO_3_ | -0.72 |  |
| Blood pH | -0.565 |  |
| Hb | -0.523 |  |
| AST |  | 0.834 |
| ALT |  | 0.806 |
| ALP |  | 0.69 |

*BUN: Blood Urea Nitrogen, Cr: Serum Creatinine, HCO_3_: Serum Bicarbonate, AST: Aspartate Transaminase, ALT; Alanine Aminotransferase, ALP: Alkaline Phosphatase.*

**Non-survivor**

|  | Principal Components | | |
| --- | --- | --- | --- |
| Variables | 1 | 2 | |
| BUN | 0.878 |  | |
| HCO_3_ | -0.82 |  | |
| Cr | 0.756 |  | |
| Blood pH | -0.63 |  | |
| K | 0.622 |  | |
| INR | 0.533 |  | |
| AST |  | | 0.958 |
| ALT |  | 0.948 | |
| ALP |  | 0.913 | |
| Consciousness |  | -0.692 | |

*BUN: Blood Urea Nitrogen, HCO_3_: Serum Bicarbonate, Cr: Serum Creatinine, K: Serum Potassium, INR: International Normalised Ratio, AST: Aspartate Transaminase, ALT: Alanine Aminotransferase, ALP: Alkaline Phosphatase.*
